# Establishment and validation of a ferroptosis-related prognostic signature for hepatocellular carcinoma

**DOI:** 10.3389/fonc.2023.1149370

**Published:** 2023-04-18

**Authors:** Yixian He, Yunyang Wu, Mengqi Song, Yanlong Yang, Yizhi Yu, Sheng Xu

**Affiliations:** ^1^ National Key Laboratory of Medical Immunology and Institute of Immunology, Naval Medical University, Shanghai, China; ^2^ Department of Traditional Chinese Medicine, The First Affiliated Hospital of Naval Medical University, Shanghai, China; ^3^ Department of Traditional Chinese Medicine, Naval Medical University, Shanghai, China

**Keywords:** drivers of ferroptosis, prognosis, hepatocellular carcinoma, immune infiltration, immunotherapy, bioinformatics methods

## Abstract

**Background:**

Hepatocellular carcinoma (HCC) is the most common type of primary liver cancer with high heterogeneity. The prognosis of HCC is quite poor and the prognostic prediction also has challenges. Ferroptosis is recently recognized as a kind of iron-dependent cell death, which is involved in tumor progression. However, further study is needed to validate the influence of drivers of ferroptosis (DOFs) on the prognosis of HCC.

**Methods:**

The FerrDb database and the Cancer Genome Atlas (TCGA) database were applied to retrieve DOFs and information of HCC patients respectively. HCC patients were randomly divided into training and testing cohorts with a 7:3 ratio. Univariate Cox regression, LASSO and multivariate Cox regression analyses were carried out to identify the optimal prognosis model and calculate the risk score. Then, univariate and multivariate Cox regression analyses were performed to assess the independence of the signature. At last, gene functional, tumor mutation and immune-related analyses were conducted to explore the underlying mechanism. Internal and external databases were used to confirm the results. Finally, the tumor tissue and normal tissue from HCC patients were applied to validate the gene expression in the model.

**Results:**

Five genes were identified to develop as a prognostic signature in the training cohort relying on the comprehensive analysis. Univariate and multivariate Cox regression analyses confirmed that the risk score was able to be an independent factor for the prognosis of HCC patients. Low-risk patients showed better overall survival than high-risk patients. Receiver operating characteristic (ROC) curve analysis confirmed the signature’s predictive capacity. Furthermore, internal and external cohorts were consistent with our results. There was a higher proportion of nTreg cell, Th1 cell, macrophage, exhausted cell and CD8^+^T cell in the high-risk group. The Tumor Immune Dysfunction and Exclusion (TIDE) score suggested that high-risk patients could respond better to immunotherapy. Besides, the experimental results showed that some genes were differentially expressed between tumor and normal tissues.

**Conclusion:**

In summary, the five ferroptosis gene signature showed potential in prognosis of patients with HCC and could also be regarded as a value biomarker for immunotherapy response in these patients.

## Introduction

Hepatocellular carcinoma (HCC) is one of the most common causes of cancer-related death worldwide with a dismal prognosis (1). The poor prognosis of HCC is associated with its late diagnosis, susceptibility to metastasis and high recurrence rates (2). Although a variety of schemes, including the primary prevention strategy, early screening and diagnosis, and more advanced treatment technologies, have been applied to the patients, the overall incidence rate and mortality of HCC continue to rise and the prognosis of HCC remains unsatisfactory (3). Thus, it is urgent to explore novel effective biomarkers to improve the prediction of prognosis and provide individualized treatment for HCC patients.

As an iron-dependent type of regulated cell death, ferroptosis is distinct from various forms of apoptosis, pyroptosis and autophagy (4). Ferroptosis was first proposed in 2012, which is characterized by excessive accumulation of lipid peroxides and reactive oxygen species (ROS) (4, 5). Typical morphological features of ferroptosis include mitochondrial abnormalities and necrosis-like changes (6). The major pathways called the exogenous and endogenous pathways regulate sensitivity of cells to ferroptosis by regulating the membrane transport protein (e.g. system XC-) and antioxidant enzymes (e.g. GPX4), respectively (7). Ferroptosis-related genes (FRGs) might be classified into 3 categories: suppressors of ferroptosis (SOFs), drivers of ferroptosis (DOFs) and others, which could act as a SOF or DOF depended on the context (8, 9). Extensive evidence has demonstrated that ferroptosis plays a crucial role in many diseases, particularly HCC (6).

Numerous studies have indicated that induction of ferroptosis showed great advantages in the treatment of malignant tumors. Sorafenib is the first approved systemic therapy for the treatment of advanced HCC patients who are not suitable for surgical resection. The mechanism of sorafenib might be to inhibit the progression of HCC by inducing ferroptosis in HCC cell (10). The drug resistance of sorafenib could be significantly ameliorated by inhibiting SOFs, like retinoblastoma (Rb), metallothinonein-1G (MT-1G) and NRF2. Haloperidol has been reported to facilitate sorafenib-induced ferroptosis by increasing the levels of Fe2^+^ and lipid peroxidation and influencing FRGs such as NRF2 and GPX4 (11). However, ferroptosis has a dual role in tumor promotion and suppression during tumorigenesis, which is dependent not only on oncogenes and tumor suppressors but also on the release of damage-associated molecular patterns (DAMPs) (7, 12). Ferroptotic damage can trigger inflammation to further promote tumor cell invasion and metastasis, and can also lead to inflammation-associated immunosuppression in the tumor microenvironment, which may contribute to tumor growth (7). For example, HMGB1 released by ferroptotic cancer cells could promote an inflammatory response in macrophages by interacting with AGER/RAGE, which could support tumor growth (12, 13). Therefore, we conducted the study to illustrate the effect of ferroptosis induced by DOFs on the prognosis of HCC.

In our study, the mRNA expression profiled and corresponding clinical data were downloaded from the public database. Afterward, we established a prognostic model consisting of five DOFs and validated it in the testing and total cohorts. The immune infiltration, immunotherapy response and chemotherapeutic drug sensitivity between the high- and low-risk groups were also compared. In addition, we conducted the quantitative Real-Time PCR (qRT-PCR) analysis to compare the expression of these five genes between tumor and normal tissues. These results implied that the five DOFs might be regarded as potential biomarkers to help predict prognosis in patients with HCC and contribute to therapeutic strategies.

## Materials and methods

### Data collection and preprocessing

**Figure 1:**
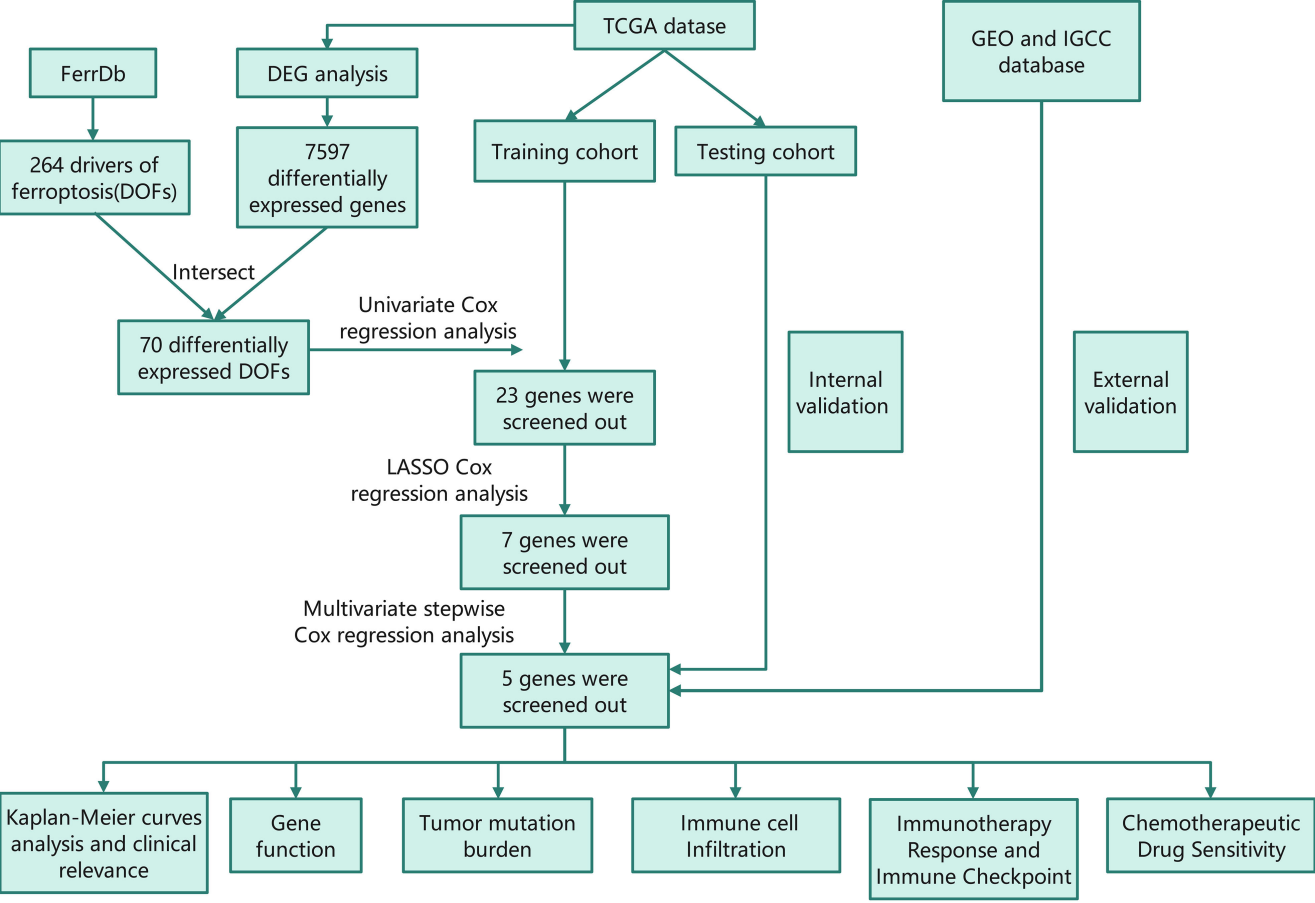
Flow chart of this study TCGA, The Cancer Genome Atlas; GEO, The Gene Expression Omnibus; ICGC, The International Cancer Genome Consortium; DEG, differentially expressed genes; DOF, driver of ferroptosis; LASSO, least absolute shrinkage and selection operator.

The RNA-sequencing data and the corresponding clinical information for HCC patients were downloaded from The Cancer Genome Atlas (TCGA) database (https://portal.gdc.cancer.gov/). The dataset, which contained 374 HCC samples and 50 normal tissue samples, was used for differential analysis. The samples with missing clinical information or overall survival (OS)< 90 days were deleted. Then, only 310 HCC samples were included in the subsequent analysis. The ferroptosis driver genes were obtained from FerrDb (http://www.zhounan.org/ferrdb/) to identify prognostic DOFs in HCC. The flow chart of this study is shown in **Figure 1**.

### Differentially expressed genes analysis

The DEGs between the tumor and normal samples were filtered by the “limma”R package in the TCGA database. The false discovery rate (FDR)>0.05 and |log2(FC)|>1 were set as the threshold. The intersecting genes between DEGs and DOFs were for further analysis.

### Construction and analysis of prognostic gene signature

We randomly divided the tumor samples into training and testing cohorts with a 7:3 ratio using the R package “caret”. Univariate regression analysis was carried out to screen the DOFs with prognostic value. LASSO analysis with “glmnet” package in R was subsequently utilized to reduce overfitting and improve the prediction significance. Finally, we could construct the optimal prognosis model using multivariate stepwise Cox regression. Univariate and multivariate Cox regression analyses were used “survival” package in R. The risk score of each patient in the training cohort could be calculated by the following formula:


$$Risk\;score=\sum_{i=1}^{n}Exp(i)\times Coef(i)$$

(Exp(i): coefficients, Coef(i): expression level, n: the number of ferroptosis driver genes)

According to the median risk score, the training set was classified into a high-risk group and a low-risk group. The OS of each group was performed by Kaplan-Meier survival curves using the “survival” package. Meanwhile, the time-dependent receiver operating characteristic (ROC) curves of 1-, 2-, and 3 years were used to evaluate the accuracy of the model. Distribution of risk score, patient survival status, and gene expression in the two risk groups was applied. In addition, univariate and multivariate regression analyses were performed to assess whether the risk model was associated with prognosis and could be regarded as the independent prognostic signature for HCC. Finally, we utilized the testing cohort and the total cohort to validate our model.

### Preliminary analysis of prognostic gene signature

Gene Ontology (GO) and Kyoto Encyclopedia of Genes and Genomes (KEGG) pathway enrichment analyses were performed to explore the functions and pathways of the intersecting genes between DEGs and DOFs. Pearson correlation analysis was used to assess the correlation among the prognostic genes. Furthermore, we examined the association between the expression of the prognostic genes and different hepatoma cell lines. Apart from the analysis of the RNA level, we also compared protein expression of the prognostic genes in normal liver and HCC tissues through immunohistochemical staining maps downloaded from The Human Protein Atlas (HPA) database (https://www.proteinatlas.org/).

### External validation of prognostic gene signature

The Gene Expression Omnibus (GEO) and The International Cancer Genome Consortium (ICGC) databases were used to confirm the model’s prognostic value. The risk scores of patients were calculated based on the same formula mentioned above, and patients were divided into high- and low-risk group according to the median risk score of GEO and ICGC cohorts, respectively. The same analyses were performed to validate the the accuracy and validity of the prognostic genes, including ROC curve analysis, Kaplan-Meier analysis, distribution of risk score, correlation between the survival time and survival status of each patient, and the expression of the prognostic genes.

### Construction and validation of nomogram

The univariate and multivariate Cox regression analyses were applied to assess the independent prognostic significance of this risk model. The nomogram was constructed using the “rms” R package, for predictive of 1, 2, and 3-year OS of HCC patients.

### Functional enrichment analysis

Gene Ontology (GO) enrichment analysis was conducted to discover the potential function of the DEGs between high- and low-risk groups. The Ensemble gene IDs were converted to official numbers using “org.Hs.eg.db”. The Gene Set Enrichment Analysis (GSEA) was a computational method, which could further determine the pathways differentially expressed between the two rick subgroups with gene set “c2.cp.kegg.v2022.1.Hs.symbols.gmt [Curated]” and “c2.cp.reactome.v2022.1.Hs.

symbols.gmt [Curated]”. NOM p-value<0.05, |NES|>1.0, and FDR<0.25 were regarded as statistically significant.

### Protein-protein interactions network

We uploaded the DEGs between high- and low-risk groups to the Search Tool for the Retrieval of Interacting Genes (STRING) online database (https://cn.string-db.org/) and constructed the interactive network of these DEGs. We chose confidence 0.4 as the screening criteria. The PPI networks were visualized using Cytoscape, and then we screened the top 10 hub genes in the PPI network using CytoHubba.

### Gene mutation analysis

We downloaded the information on genetic alterations from the TCGA cohort and calculated the TMB score of each patient in the TCGA cohort with the mutation data. The differences in TMB between the high- and low-risk groups and the relationship between TMB and survival rates were also explored. Meanwhile, the “Maftools” package of R was utilized to analyze the quantity and quality of gene mutations in the two risk subgroups. We evaluated the mutations of the prognostic genes in HCC and detected the main type of mutation as well. Then, correlation analysis was performed between the types of major mutations in the prognostic genes and mRNA expression.

### Immune cell infiltration analysis

The infiltration levels of immune cells were obtained from a specific website (http://bioinfo.life.hust.edu.cn/ImmuCellAI#!/ run with the old version). Subsequently, we compared the composition of immune cell infiltration between high- and low-risk groups and examined the association between the prognostic risk scores and the immune microenvironment.

### Analysis of immunotherapy response and immune checkpoint genes

Immune checkpoint inhibitors (ICIs) is an effective treatment strategy against a variety of tumors. Several well-known immune checkpoints genes were retrieved from previous articles (14–17), and were compared in risk subgroups. In addition, we have obtained some information of ICGs learned from relevant article, and the ICGs were splited into two groups according to the result of the article (18). We firstly compared the expression of these ICGs in the two risk groups, and further explored the innate relationship between these ICGs and the 5 genes. Tumor Immune Dysfunction and Exclusion (TIDE) algorithm is a computational prediction tool which could explore the performance of the given prognostic signature in predicting the response of patients to immunotherapy. Then, we calculated TIDE score by using TIDE website (http://tide.dfci.harvard.edu/) to assess the immunotherapeutic sensitivity of HCC patients.

### Chemotherapeutic drug sensitivity analysis

The “pRRophetic” package was employed to pick out the targeted drugs and to figure out which drugs were significantly correlated with the risk score. Semi-inhibitory concentrations (IC50) were calculated to act as the outcome and compared by Wilcoxon sign-rank test.

### Tissue sample collection

A total of 10 pairs of HCC tissues samples and paracancerous tissues samples were obtained from patients who had undergone hepatocellular carcinoma resection in the Eastern Hepatobiliary Surgery Hospital during February 1, 2023 to February 20, 2023 from the group of one professor. None of them received radiotherapy or chemotherapy before surgery. All patients provided written informed consent.

### Quantitative real-time PCR analysis

Total RNA from HCC and normal liver tissue samples was extracted using Trizol reagent. Total RNA was reversed to cDNA by M-MLV Reverse Transcriptase (TaKaRa). Then, qRT-PCR was performed using SYBR Premix ExTaq Kit (TaKaRa) on QuantStudio 7 Flex system to amplify cDNA with specific primers. β-actin was used as internal standard control, respectively. The relative expression levels were determined by 2^-ΔΔCt^. The primer sequences are listed as in **Table 1**.

**Table 1:** Primer sequences of genes for qPCR.

Gene	Primer sequence (5’ to 3’)
NRAS	F: ATGACTGAGTACAAACTGGTGGT R: CATGTATTGGTCTCTCATGGCAC
HRAS	F: ATGACGGAATATAAGCTGGTGGT R: GGCACGTCTCCCCATCAATG
β-actin	F: ACAATGAGCTGCTGGTGGCT R: GATGGGCACAGTGTGGGTGA

### Statistical analysis

All statistical data were analyzed by Strawberry Perl (5.30.0.1) and R software (R version:4.1.0). Multiple R packages, such as limma, survival, caret and so on, were adopted in this study. Statistical significance was set at probability values of p<0.05. *p<0.05; **p<0.01; ***p<0.001 

## Results

### Identification of differentially expressed genes

**Figure 2:**
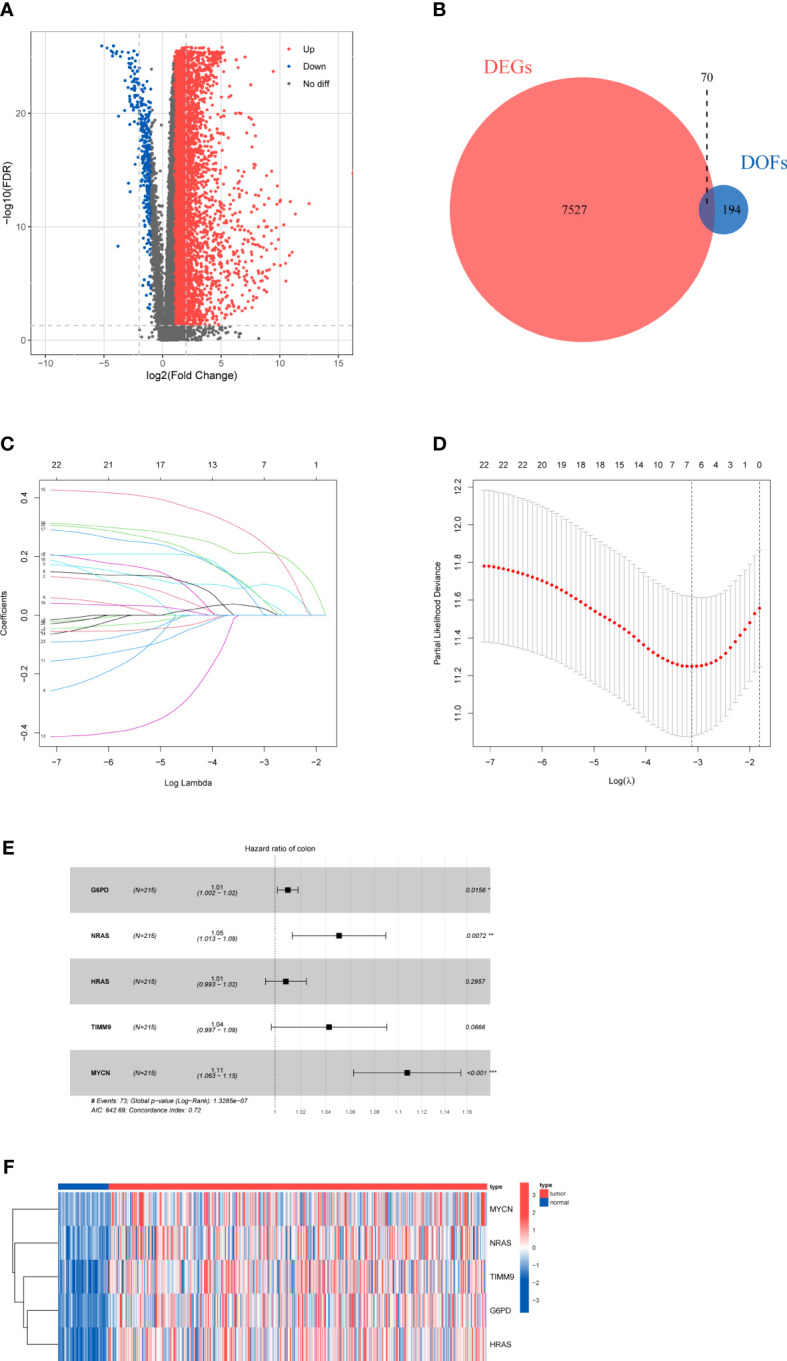
Results of differential gene analysis **(A)** Volcano map of the expression of ferroptosis-related genes in normal and tumor samples. **(B)** Venn diagram of the differentially expressed genes and DOFs. **(C)** Lasso coefficient profiles of 23 DOFs. **(D)** Tuning parameter in the lasso model. **(E)** Five DOFs were finally identified by multivariate cox regression. **(F)**The heatmap of five-gene signature expression.

A total of 424 samples, which containing 50 normal samples and 374 HCC samples, were downloaded from the TCGA database. After the difference analysis, 7597 DEGs between the tumor and normal samples were identified (**Figure 2A**). Moreover, 264 DOFs were obtained from FerrDb. After intersecting differentially expressed genes and DOFs, we screened out 70 differentially expressed DOFs (**Figure 2B**).

### Development and validation of the prognostic signature

According to the inclusion criteria, only 310 HCC samples were included in the subsequent analysis. We randomly divided HCC samples into training cohort and testing cohort with a 7:3 ratio. After merging integrated gene expression profiles, 304 samples were ultimately enrolled.

To explore the prognostic genes for OS of HCC, we performed the univariate Cox regression analysis with the 70 DOFs in the training cohort. The result showed that 23 DOFs were statistically significant (p<0.05) and might have prognostic value. Subsequently, we obtained 7 DOFs using a LASSO Cox regression analysis to reduce overfitting and improve the prediction significance (**Figures 2C, D**). Finally, a five-gene signature which consisted of G6PD, HRAS, NRAS, TIMM9 and MYCN was identified *via* multivariate stepwise Cox regression (**Figure 2E**). A heatmap between normal and tumor tissues showed the expression of G6PD, HRAS, NRAS, TIMM9, and MYCN (**Figure 2F**). Among these 5 DOFs, all of them were risk genes for the prognosis of HCC patients. We calculated the risk score through the following formula:


$$\begin{array}{c} Risk\;Score=G6PD\\ {}*0.010086754+NRAS*0.051905262\\ +HRAS*0.012261478+TIMM9\\ {}*0.039085391+MYCN*0.106958042 \end{array}$$

**Figure 3:**
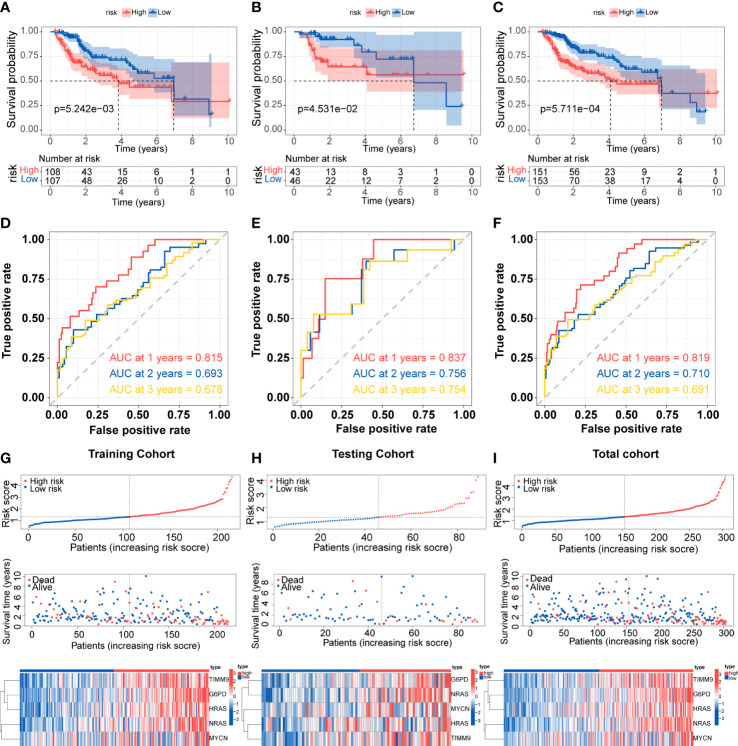
Development and validation of the prognostic signature **(A-C)** Kaplan-Meier survival for overall survival in the two subgroups for the training cohort, testing cohort, and total cohort. **(D-F)** ROC curve evaluated the prognostic value of the risk model in the training cohort, testing cohort, and total cohort. **(G-I)** Distribution of risk score, correlation between the survival time and survival status of each patient, and the heatmaps of this signature expressions in the training cohort, testing cohort, and total cohort.

Patients in the training cohort were divided into low-risk group and high-risk group based on the median value of risk scores. The Kaplan-Meier curves revealed that patients in the high-risk group were significantly relevant to worse overall survival (**Figure 3A**). Similar results were also verified in the testing cohort and total cohort (**Figures 3B, C**). ROC curves were generated to evaluate the capability of the prognostic significance. The AUC scores in the training cohort were 0.815, 0.693, and 0.678, and the cut-off values were 1.714, 2.012, and 2.012 for 1-, 2-, and 3 years, respectively (**Figure 3D**). For the testing cohort, the AUC scores for the 1-, 2-, and 3 years reached 0.837, 0.756, and 0.754, and the cut-off values were 1.278. (**Figure 3E**). The total cohort had high AUC scores which were all above 0.69, and the cut-off values were 1.714, 2,050, and 1.704 for 1-, 2-, 3 years, respectively (**Figure 3F**). The risk score, survival time and survival status, and gene expression of the five genes in the training cohort and both validation cohort are shown in **Figures 3G-I** which revealed that the high-risk group had more death cases and higher expression levels of the five genes. Overall, these results indicated the accuracy and robustness of our prognostic model.

### Relationship between the prognostic signature and clinical features

**Figure 4:**
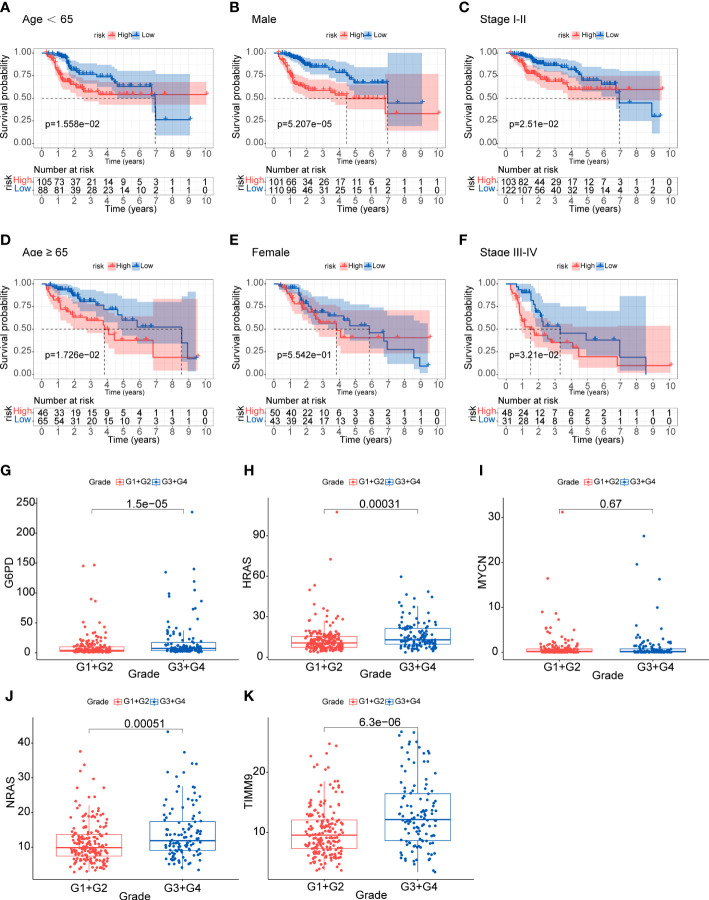
Analysis of clinical relevance and risk score **(A-F)** Analysis of relationship between clinical features and risk score. **(G-K)** Analysis of the five genes in different tumor grades.

We assessed the prognostic significance of the risk model in the TCGA total cohort with different subgroups of clinical features. High-risk patients regardless of age and stage all showed worse OS (p<0.05) (**Figures 4A-F**). The stratified analysis results confirmed the model’s applicability in the subgroups. We also analyzed the expression of the five genes in different tumor grades. The results showed that most genes were up-regulated in G3 and G4, except MYCN (**Figures 4G-K**). This indicated that the prognostic signature was significantly correlated with tumor grade.

### Preliminary analysis of prognostic gene signature

We firstly used the GO and KEGG analyses to investigate the potential functions and pathways of these 70 DOFs. According to GO analysis, the 70 DOFs were mainly enriched in metabolic process, and we also found that the reactive oxygen species metabolic process was significantly important to G6PD and MYCN (**Supplementary Figures 1A, B**), and TIMM9 may participate in protein-related pathways like protein carrier activity in the GO analysis (**Supplementary Files**). Based on the KEGG analysis, G6PD was enriched in central carbon metabolism in cancer, and HRAS and NRAS could be involved in many signaling pathways, such as AGE-RAGE signaling pathway in diabetic complications, human cytomegalovirus infection and renal cell carcinoma (**Supplementary Figures 1C, D**). Then, theresults of the Pearson correlation analysis showed that there was weak correlation among G6PD, HRAS and TIMM9 (**Supplementary Figure 1E**). The levels of gene expression in different hepatoma cell lines were shown in **Supplementary Figures 2A-E**.To further confirm the protein expression characteristics of the 5 genes, we obtained the immunohistochemical data of G6PD, HRAS, NRAS and TIMM9 from the HPA database. In the HPA database we did not find the information of MYCN at protein level. Nevertheless, the immunohistochemical staining demonstrated that the protein expression of the other 4 genes in HCC tissues was higher than that in normal tissues (**Supplementary Figure 3**), which was consistent with their mRNA expression (**Figure 2F**).

### External validation of prognostic gene signature

**Figure 5:**
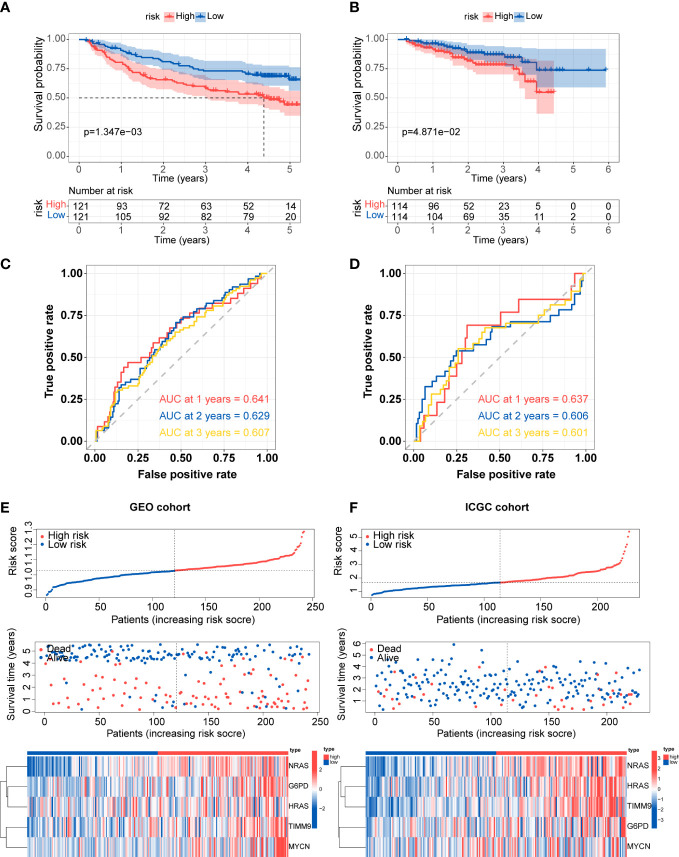
External validation of prognostic gene signature **(A, B)** Kaplan-Meier survival for overall survival in the two subgroups for the GEO cohort and ICGC cohort. **(C, D)** ROC curve verified the prognostic value of the risk model in the GEO cohort and ICGC cohort. **(E, F)** Distribution of risk score, correlation between the survival time and survival status of each patient, and the heatmaps of this signature expressions in the GEO cohort and ICGC cohort.

The predictive efficacy of the five-gene signature was validate in the two external cohorts, GEO (GSE14520) and ICGC cohorts. Similarly, the Kaplan-Meier curves demonstrated that patients in the high-risk group exhibited poorer overall survival than those in the low-risk group (**Figures 5A, B**). According to ROC analysis, the AUC scores in the GEO cohort at 1, 2, and 3 years were 0.641, 0.629, and 0.607, respectively (**Figure 5C**). For the ICGC cohort, the AUC scores for the 1-, 2-, and 3 years reached 0.637, 0.606, and 0.601 (**Figure 5D**). Consistent with TCGA cohort, the distribution of risk score and survival status of each patient showed the same trends in GEO and ICGC cohort, and the expression of G6PD, HRAS, NRAS, MYCN and TIMM9 increased in high-risk group (**Figures 5E, F**).

### Construction and validation of nomogram

**Figure 6:**
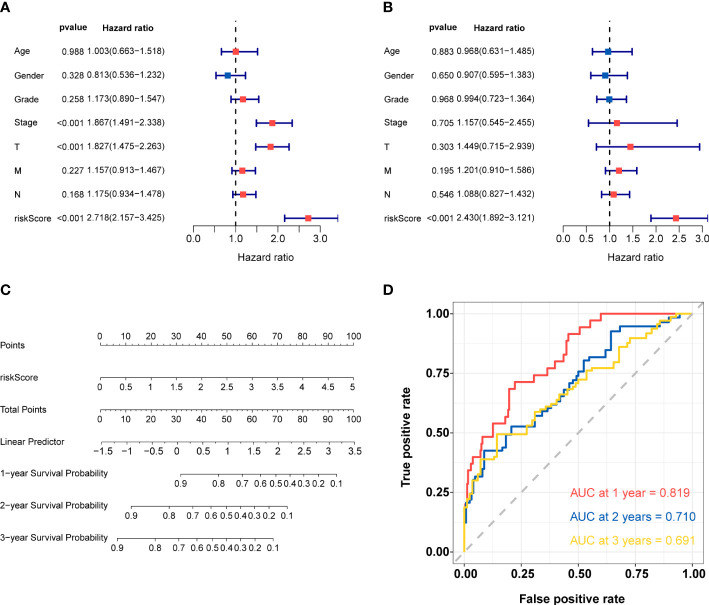
Construction and validation of nomogram **(A)** Univariate Cox regression analysis for risk score and other clinic pathological factors. **(B)** Multivariate cox regression analysis for risk score and other clinic pathological factors. **(C)**The nomogram for predicting 1-, 2-, and 3-years overall survival by risk score. **(D)** ROC curve analysis of nomogram according to the 1-, 2-, and 3-year overall survival.

Univariate and multivariate Cox regression analyses were utilized to confirm whether the risk score and other clinic pathological factors could be the independent factor for the prognosis of HCC. The results of univariate Cox analysis showed that stage, pathologic T and risk score were significantly correlated with OS (p<0.05) (**Figure 6A**). Meanwhile, only risk score showed similar result in multivariate Cox analysis (**Figure 6B**). These results suggested that the risk score could be used as the independent prognostic factor for HCC. According to the results of univariate and multivariate analyses in the TCGA-total cohort, risk score was considered to establish the nomogram (**Figure 6C**). The AUC of 1-, 2-, and 3-year overall survival predictions were 0.819, 0.710, and 0.691, respectively (**Figure 6D**). Above results validated the accuracy of nomogram in predicting prognosis for HCC patients.

### Functional enrichment analysis

**Figure 7:**
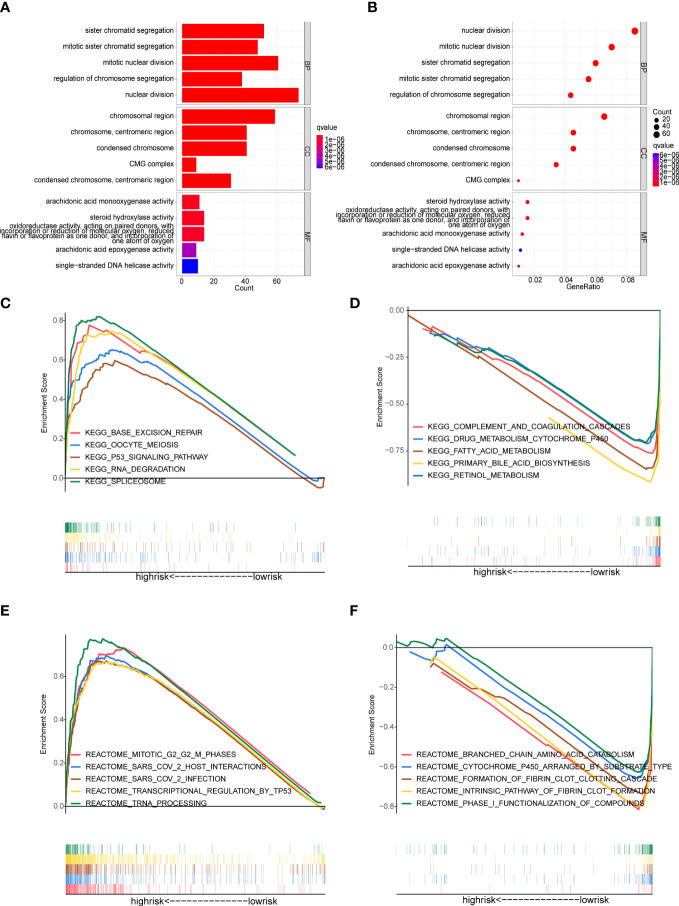
Functional enrichment analysis **(A, B)** GO enrichment analysis of the DEGs between the high- and low-risk groups. **(C-F)** Gene Set Enrichment Analysis (GSEA). **(C, D)** Enriched KEGG pathways in the high-risk and low-risk groups. **(E, F)** Enriched Reactome pathways in the high-risk and low-risk groups.

The GO-biological process (GO-BP) enrichment analysis showed that these DEGs between high- and low-risk groups were mainly enriched in nuclear division, mitotic nuclear division and sister chromatid segregation. In GO-cellular component (GO-CC) analysis, these DEGs were significantly enriched in chromosomal region, chromosome, centromeric region and condensed chromosome. For the GO-molecular function (GO-MF) analysis, these DEGs were associated with steroid hydroxylase activity, arachidonic acid monooxygenase activity etc (**Figures 7A, B**).

GSEA analysis was applied to detect the underlying pathways in HCC between the high- and low-risk groups. The KEGG pathway enrichment analysis showed that the genes in the high-risk group were significantly enriched in spliceosome, base excision repair, RNA degradation and oocyte meiosis (**Figure 7C**). Meanwhile, patients in the high-risk group were also involved in tumor-related signaling pathways like P53 (**Figure 7C**). In addition, multiple metabolic processes that involved more physiological functions of the liver were enriched in the low-risk group, including complement and coagulation cascades, primary bile acid biosynthesis, drug metabolism cytochrome P450, fatty acid metabolism and retinol metabolism (**Figure 7D**). Moreover, the GSEA analysis, along with reactome pathways, revealed that pathways correlated with the high-risk group were mainly focused on transcriptional regulation and SARS-CoV-2 (including transcriptional regulation by TP53, tRNA processing, SARS-CoV-2 infection and so on) (**Figure 7E**). In the low-risk group, the enriched reactome pathways were also involved in metabolic processes, such as branched chain amino acid catabolism, cytochrome P450 arranged by substrate type and phase I functionalization of compounds (**Figure 7F**).

### Construction of protein-protein network

**Figure 8:**
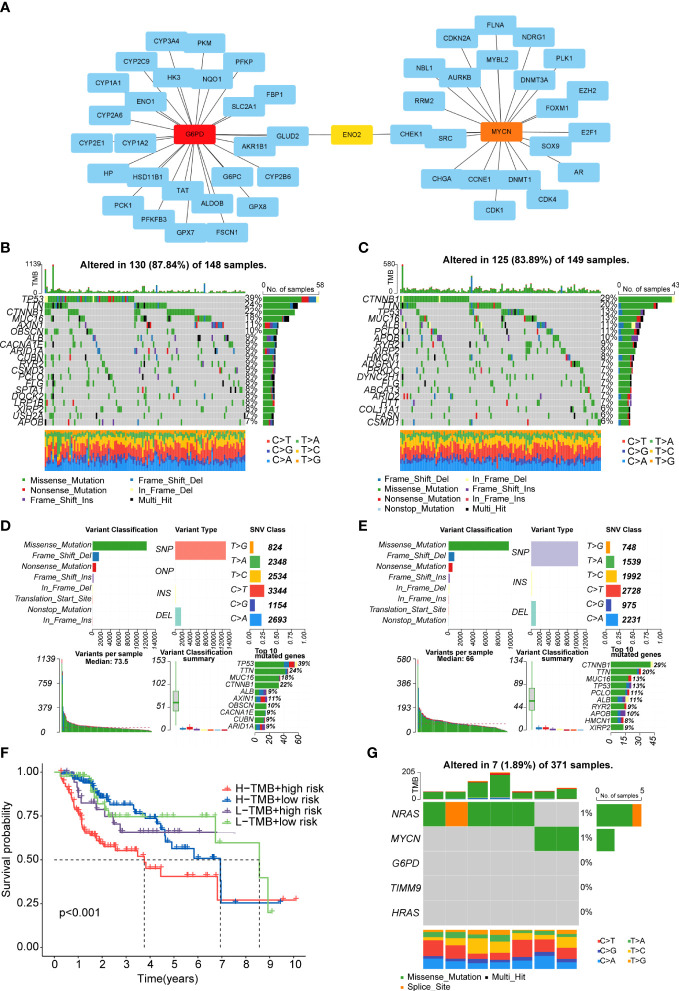
PPI network and tumor mutation analysis **(A)** The PPI network of G6PD and MYCN. **(B, C)** Waterfall plot of detailed mutation information of top 20 genes in high-risk **(B)** and low-risk **(C)** groups. **(D, E)** Mutation information distinguished by different classification categories in high-risk **(D)** and low-risk **(E)** groups. **(F)**. The K-M survival curve shows the combined effect of TMB and risk score on the OS. **(G)** Mutation information of the prognostic genes.

Firstly, we obtained the DEGs between high- and low-risk groups. The PPI network of these DEGs was constructed basing on the STRING database and the Cytoscape software, and then we screened the top 10 hub genes in the PPI network (**Supplementary Figures 4A, B**). It was regretted that only two (G6PD and MYCN) of the five genes which we have identified were in the DEGs between the two risk groups. Therefore, we used the CytoHubba function of Cytoscape software to find the hub genes of G6PD and MYCN. The results showed that ENO2 was the intersection of G6PD and MYCN (**Figure 8A**). The overall survival analysis of ENO2 was performed using Kaplan-Meier curve, which validate the prognostic value of ENO2 (**Supplementary Figure 4C**).

### Gene mutation analysis

After downloading the mutation data from the TCGA cohort, we explored the differences in TMB and survival rates in the two sub-risk groups. Unfortunately, there was no significant difference in TMB between the high- and low-risk groups and the relationship between TMB and survial rates was not obvious (**Supplementary Figures 4D, E**). However, patients in the high TMB and high-risk group had the worst prognosis than the other groups (**Figure 8F**). The waterfall plot was performed to exhibit the top 20 genes with the high frequency of alteration in the two risk subgroups (**Figures 8B, C**).Moreover, in these two groups, missense mutations, single-nucleotide polymorphism (SNP), and C>T mutation were the main mutation type of different classification categories, respectively (**Figures 8D, E**). We also exhibit the median variation and variant types (**Figures 8D, E**). In the whole samples, the mutation rates of the five prognostic genes were not high, only NRAS and MYCN could mutate (**Figure 8G**). Missense mutations was also the main type (**Figure 8G**).

### Correlation between risk score and immune microenvironment

**Figure 9:**
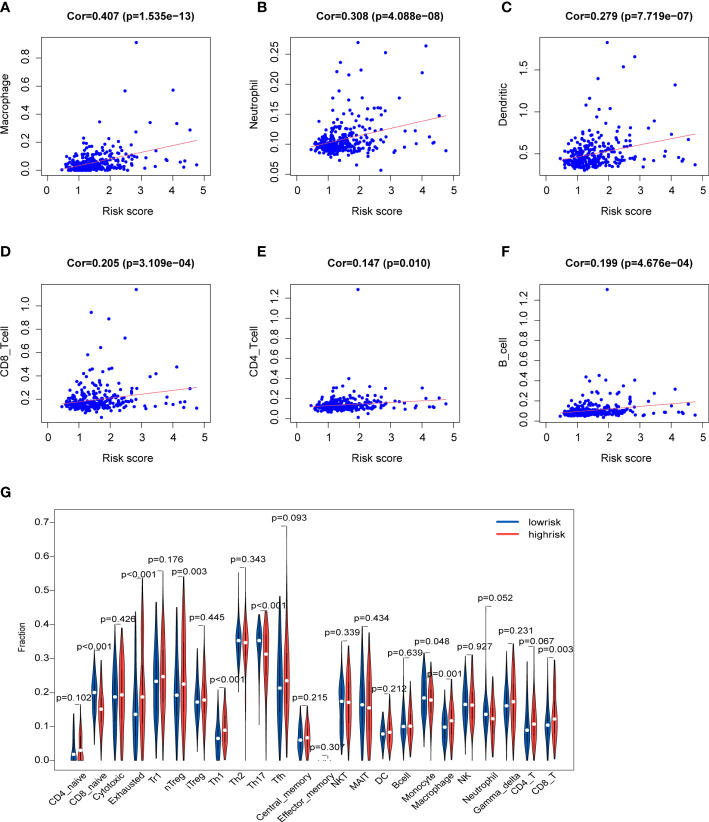
Characteristics of immune microenvironment **(A-F)** Relationships between the risk score and infiltration abundances of six types of immune cells. **(G)** Comparison of immune cell proportions.

Immune cells in the tumor microenvironment profoundly affect the biological behavior of the tumor (19, 20). The relationship of the prognosis model with immune cell infiltration was investigated to evaluate whether the risk score partly reflected the tumor immune microenvironment(TIME) status. The results indicated that neutrophils (Cor=0.308; p=4.088e-08), macrophages (Cor=0.407; p=1.535e−13), DCs (Cor=0.279; p=7.719e-07) and CD8^+^T cells (Cor=0.205; p=3.109e-04) contents showed association with high-risk group (**Figures 9A-D**). However, CD4^+^T cells (Cor=0.147; p=0.01) and B cells (Cor=0.199; p=4.676e-04) had not marked relationship with risk score (**Figures 9E, F**). Besides, the infiltration levels of immune cells were obtained online. HCC patients belonging to the high-risk group had higher proportions of immune cells including nTreg cell, Th1 cell, macrophage, exhausted cell and CD8^+^T cell, while the proportions of naive CD8^+^T cell, Th17 cell and monocyte were lower in the high-risk group (**Figure 9G**).

### Immunotherapy response and immune checkpoint

**Figure 10:**
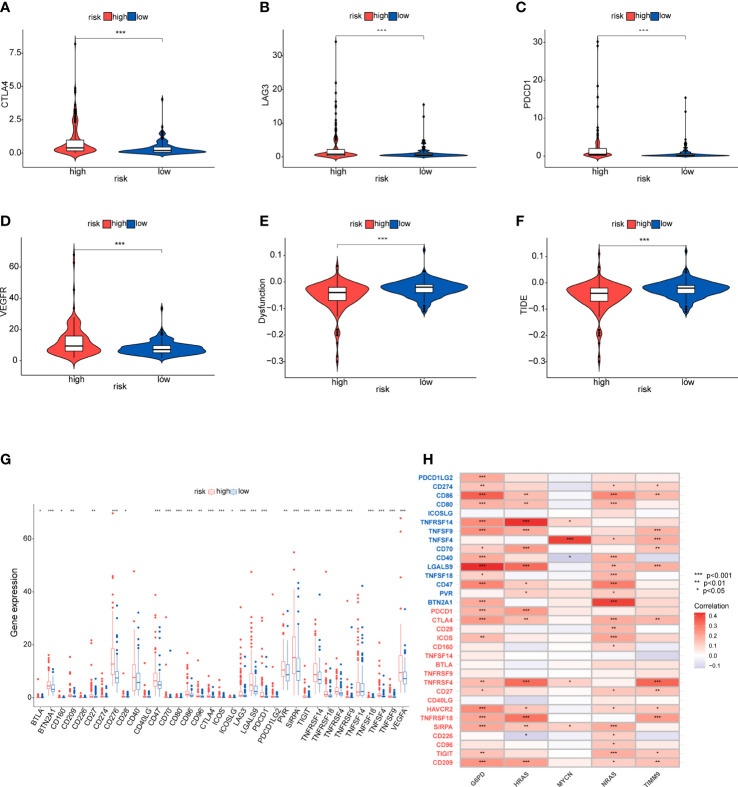
Immunotherapy response and immune checkpoint in the two risk groups **(A-D)** Comparison between the expression of four immune checkpoints in the high- and low-risk groups. **(E, F)** Comparison of dysfunction score and TIDE score. **(G, H)** The correlation between the five-gene signature and immnue checkpoint genes. Tumor cell/APC/DC groups genes (blue), T cell group genes (red).

Considering the clinical importance of immunotherapy, we then explored the association between the risk score and four immune checkpoint genes (PDCD1, CTLA4, LAG3 and VEGFA). The results showed a statistically significant difference in the expression of immune checkpoints which was higher in the high-risk group (**Figures 10A-D**). Then, some ICGs obtained from an article were made a comparison in the two risk groups. These ICGs were divided into T cell goups and Tumor cell/APC/DC group. Likewise, the expression of these ICGs was higher in the high-risk group whether in T cell or Tumor cell/APC/DC group (**Figure 10 G**). We also found that G6PD, NRAS and HRAS may have a positively moderate correlation with most of IGCs in Tumor cell/APC/DC group than in T cell group (**Figure 10H**). To further evaluate the responses of patients to immunotherapy, we used the TIDE algorithm, and patients with lower TIDE score may benefit from immunotherapy. Dysfunction score and TIDE score were lower in the high-risk group, suggesting that high-risk patients could respond better to immunotherapy (**Figures 10E, F**).

### Chemotherapeutic drug sensitivity

**Figure 11:**
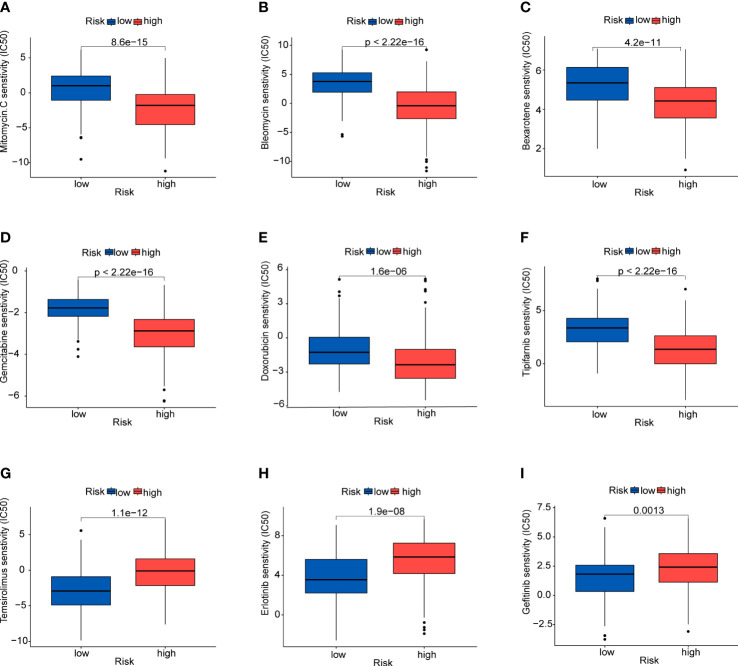
Prediction of sensitivity to certain drugs **(A)** Mitomycin **(C, B)** Bleomycin. **(C)** Bexarotene. **(D)** Gemcitabine. **(E)** Doxorubicin. **(F)** Tipifarnib. **(G)** Temsirolimus. **(H)** Erlotinib. **(I)** Gefitinib.

To identify the potential drugs for HCC, we analyzed the efficacy of chemotherapeutic drugs by comparing IC50 values between the high- and low-risk group (**Figure 11**). Results showed that patients in the high-risk group had lower IC50s of mitomycin C, bleomycin, bexarotene, gemcitabine, doxorubicin and tipifarnib than those in low-risk group,which means these chemotherapeutic drugs may have a good effect on patients in the high-risk group. Whereas the IC50s of temsirolimus, erlotinib and gefitinib were higher in the high-risk group.

### The mRNA relative expression of genes in the risk model

**Figure 12:**
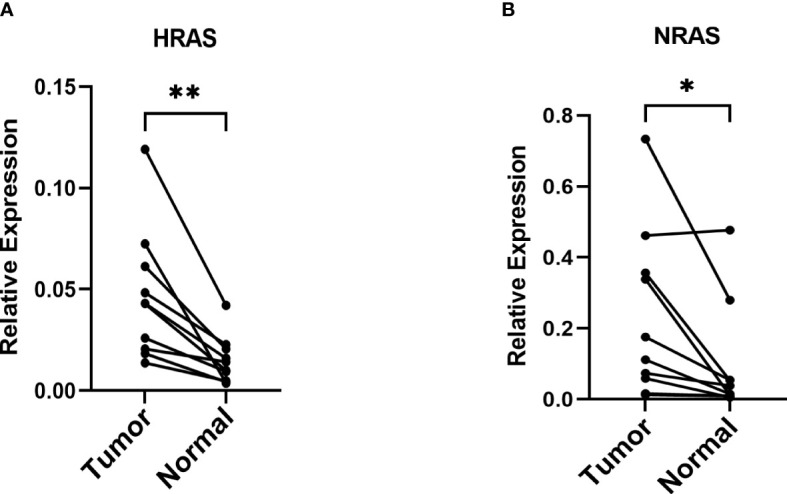
The mRNA relative expression of genes in the risk model **(A, B)** The mRNA relative expression of HRAS and NRAS in paired tumor tissues with β-actin as an internal control. *P< 0.05; **P< 0.01.

Because the expression of NRAS and HRAS were significantly different between Grade1+2 and Grade3+4, we selected these two genes to perform the qPCR. The β-actin was exploited as an internal control, and the results showed that NRAS and HRAS were expressed differentially between HCC and paracancerous tissues (**Figures 12A, B**).

## Discussion

Although landmark advances in HCC prevention, early screening and treatment options, HCC is still one of the most common malignant disease with a high mortality and a poor prognosis, accounting for more than 500000 deaths each year (20–22). In our country, the incidence of HCC is mainly concentrated in people aged 30-60 and the five-year survival rate remains low, only 14.1% (23, 24). In order to improve the OS of HCC patients, it is necessary to search novel and effective markers of prognosis. Ferroptosis has been regarded as a novel form of regulated cell death, which could be closely related to HCC tumorigenesis and progression. Regulation of iron metabolism and ROS accumulation may affect the occurrence of ferroptosis. Compared with normal cells, tumor cells exhibit higher intracellular iron storage to facilitate their growth and proliferation, but the excessive iron could also cause tumor cells more sensitive to ferroptosis (25). Some genes are well-defined as DOFs which could promote ferroptosis. However, the effect of these DOFs on prognosis of HCC patients has been unclear and required comprehensive analyses.

In this study, we constructed a five-gene signature which consisted of G6PD, HARS, NARS, TIMM9 and MYCN by univariate Cox, LASSO-Cox and multivariate stepwise Cox regression. This signature had an effectively and stably predictive performance both in the training and testing cohort. High-risk patients presented with worse overall survival. According to corresponding analyses, the signature could be considered as an independent factor for the prognosis of HCC. The data from GEO and ICGC databases were used as validation cohorts to evaluate the reliability and accuracy of this five-gene signature. Meanwhile, we verified the expression of NRAS and HRAS which are consisted of the prognostic model by qPCR. The qPCR results revealed that these genes had the tendency of increasing their expression in HCC and could affect the development of HCC.

With regard to the five DOFs, numerous studies have demonstrated their crucial roles in tumor development. The expression of G6PD was elevated in many cancers, including HCC, which was positively correlated with tumors proliferation, migration and invasion (26, 27). G6PD could affected many enzymes, like NADPH, which is important for ROS production (26). Ras protein (HRAS and NRAS) are common oncogenes and mutant RAS could be considered as a driver of tumor initiation and maintenance (28). The RAS-RAF-ERK-pathway has a critical role in human tumor occurrence. Sorafenib and regorafenib are the effective therapeutic methods for advanced HCC, which target multiple kinase-related pathways including the RAS-RAF-ERK-pathway in HCC cells (29). HRAS and NRAS were proven to be correlated to the prognosis of HCC. NRAS contributes to sorafenib resistance and NRAS knockdown might partially restore the effect of sorafenib (29). TIMM9 is a mitochondrial protein whose expression is increased in various cancers, including thyroid, lung and liver cancer (30). MYCN belongs to MYC family which has been proved to be a biomarker for HCC and a valuable target for anti-HCC therapy (31). Lipid biosynthesis has been confirmed to be important for MYCN-derived tumors and silencing of MYCN could inhibit cell proliferation and promote cell death in HCC cells (32).

To reveal the biological functions and molecular mechanism of the DEGs between the high- and low-risk group and the five DOFs, we further conducted GO and GSEA analysis. The GO analysis showed these DEGs may be related to cell division. The KEGG pathways, including spliceosome, base excision repair, RNA degradation, oocyte meiosis and P53 signaling pathway, were significantly enriched in high-risk group. Some pathways are closely associated with cell cycle and tumor progression. The “spliceosome” pathway were the most enriched in high-risk group. Previous study showed that genes in the spliceosome pathway were upregulated in tumor tissue and these genes had influence on HCC progression (33). Furthermore, the GSEA analysis, along with reactome pathways, showed that the enriched pathways were involved transcriptional regulation and SARS-CoV-2 in the high-risk group. From KEGG and reactome analyses, we found that TP53 is a critical gene for patients in the high-risk groups. Study has indicated that the infective SARS-CoV-2 is related to immune escape through different approaches like IFN-1 production dysregulation and cytokines related immune escape (34).

Immunotherapy has become a new strategy of treatment and offered survival benefits for HCC patients around the world (35). The clinical researches of immune checkpoint are relatively mature and adequate. Regulation of immune checkpoints has gradually become the widely used form of immunotherapy (36). Additionally, current studies have revealed that TMB may become a potential predictive biomarker for immunotherapy, and high TMB may be correlated with good response of immune checkpoint inhibitor (ICI) therapy (37–39). Firstly, we studied gene mutations in the high- and low-risk groups, and analyzed mutations of the prognostic genes in HCC. The results showed no statistical difference in TMB between the high and low-risk groups, and TMB had an unobvious effect on the survival rates. However, we found that missense mutations were the common type, and TP53 and CTNNB1 were the predominat mutated genes in the two groups. It is well known that TP53 and CTNNB1 are frequently mutated in various cancers. TP53 has been shown to be a tumor suppressors in regulating metabolism of tumor cells (40, 41). Nevertheless, HCC tissues with TP53 mutation could detect the vascular invasion and angiogenesis, and these tissues are characterized by hypodifferentiation (42, 43). CTNNB1 gene encodes the protein β-catenin which has been found could promote immune escape and may affect the immunotherapy in HCC (44). Moreover, better understanding of the immune microenvironment will help to develop new methods to treat HCC. According to immune infiltration analysis, there were significant difference in multiple immune cells between the two groups identified by the risk scores. We found the patients in high-risk group had higher proportion of nTreg cell, Th1 cell, macrophage, exhausted cells and CD8^+^T cells, while the proportions of naive CD8^+^T cell, Th17 cell and monocyte were lower. Treg cell could enhance immunosuppressive environment and play a role in tumor progression, which has been well documented in many cancers, including HCC (45). Macrophages could promote cancer stem cells by secreting IL-6 and activating of STAT3 signaling and subsequently contribute to tumor growth (46). Above results suggested that the poor prognosis in the high-risk group may due to the immunosuppressive environment. Besides, based on the immune checkpoint inhibitors (ICIs), immunotherapy offers great promise in the treatment of HCC. We detected the immune checkpoints between the two risk groups. Higher expression of PDCD1, CTLA4, LAG3 and VEGFA was exhibited in the high-risk group, suggesting the high-risk patients may benefit from ICIs. In addition, patients in high-risk group showed a lower dysfunction score and TIDE score, suggesting that these patients may have the better responses to immunotherapy. Finally, we evaluated the drug sensitivity using the “pRRophetic” package that indicated potential benefits from chemotherapy for patients.

This study was to explore the function of DOFs in HCC prognosis, which screened out a five-gene signature with potential values in predicting the prognosis of patients with HCC. Besides, our study has demonstrated that immunotherapy is more appropriate for patients in the high-risk group, and some effective drugs for patients were also selected. There exited certain limitations in this study. We validate the study with clinical experiment, while the experimental result is not abundant and more samples are required for further validation. In addition, the specific role of the five genes in ferroptosis and HCC need to conduct experiments in the future.

## Conclusion

We have constructed and verified a five-gene signature consisted of G6PD, HRAS, NRAS, TIMM9, and MYCN that can predict accurately. The five-gene signature could be regarded as a potential biomarker and provide the possibility to predict the prognosis for HCC patients. The prognostic model also profoundly impacts tumor immunity, immunotherapy, and drug sensitivity.

## Data availability statement

The datasets presented in this study can be found in online repositories. The names of the repository/repositories and accession number(s) can be found in the article/**Supplementary Material**.

## Ethics statement

The studies involving human participants were reviewed and approved by the Institute Research Ethics Committees of Navy Medical University(Second Military Medical University). The patients/participants provided their written informed consent to participate in this study. Written informed consent was obtained from the individual(s) for the publication of any potentially identifiable images or data included in this article.

## Author contributions

YH and YW conceived of the study and drafted the manuscript. MS participated in the bioinformatic analyses. YH and YW conducted the experiment. YLY, YZY and SX supervised the study and offered guidance. YH and YW contributed equally to this article. All authors contributed to the article and approved the submitted version.
