# Supplementary figures and images for: Establishment and validation of a ferroptosis-related prognostic signature for hepatocellular carcinoma
Source: Front Oncol. 2023 Apr 18;13:1149370. doi: 10.3389/fonc.2023.1149370 (PMC10151679; doi:10.3389/fonc.2023.1149370)

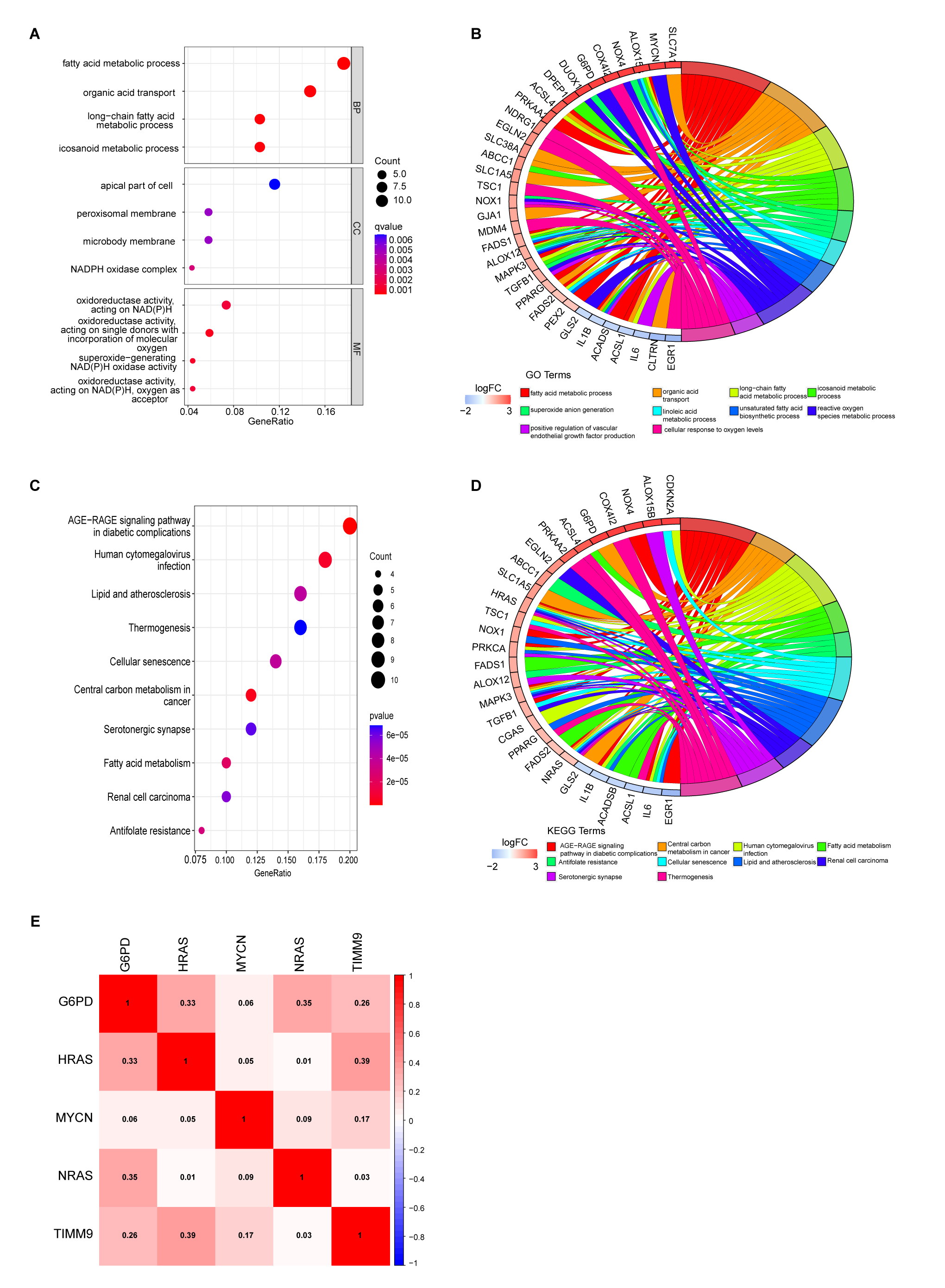

Supplement: Supplementary file 1 [file Image_1.tif]

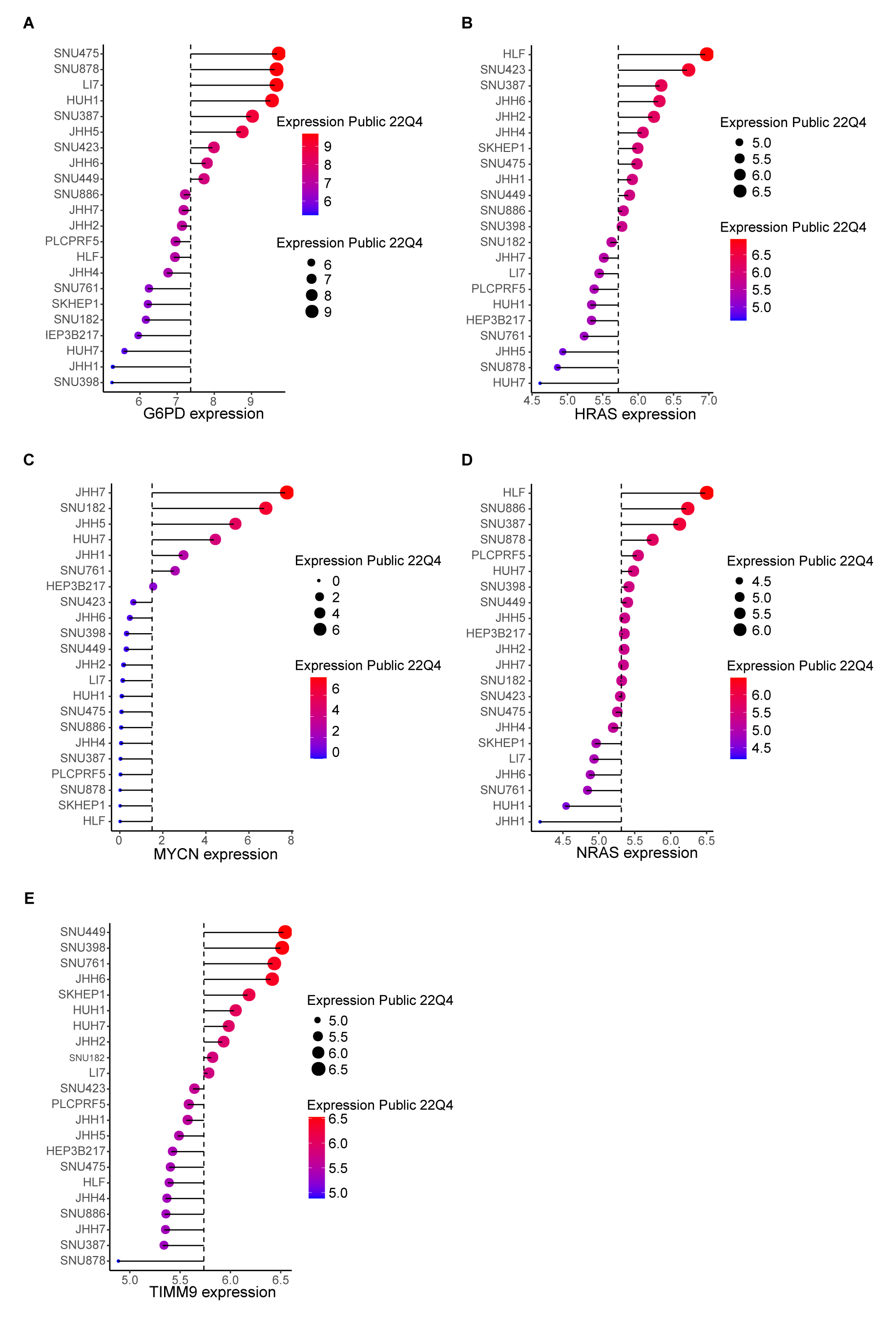

Supplement: Supplementary file 2 [file Image_2.tif]

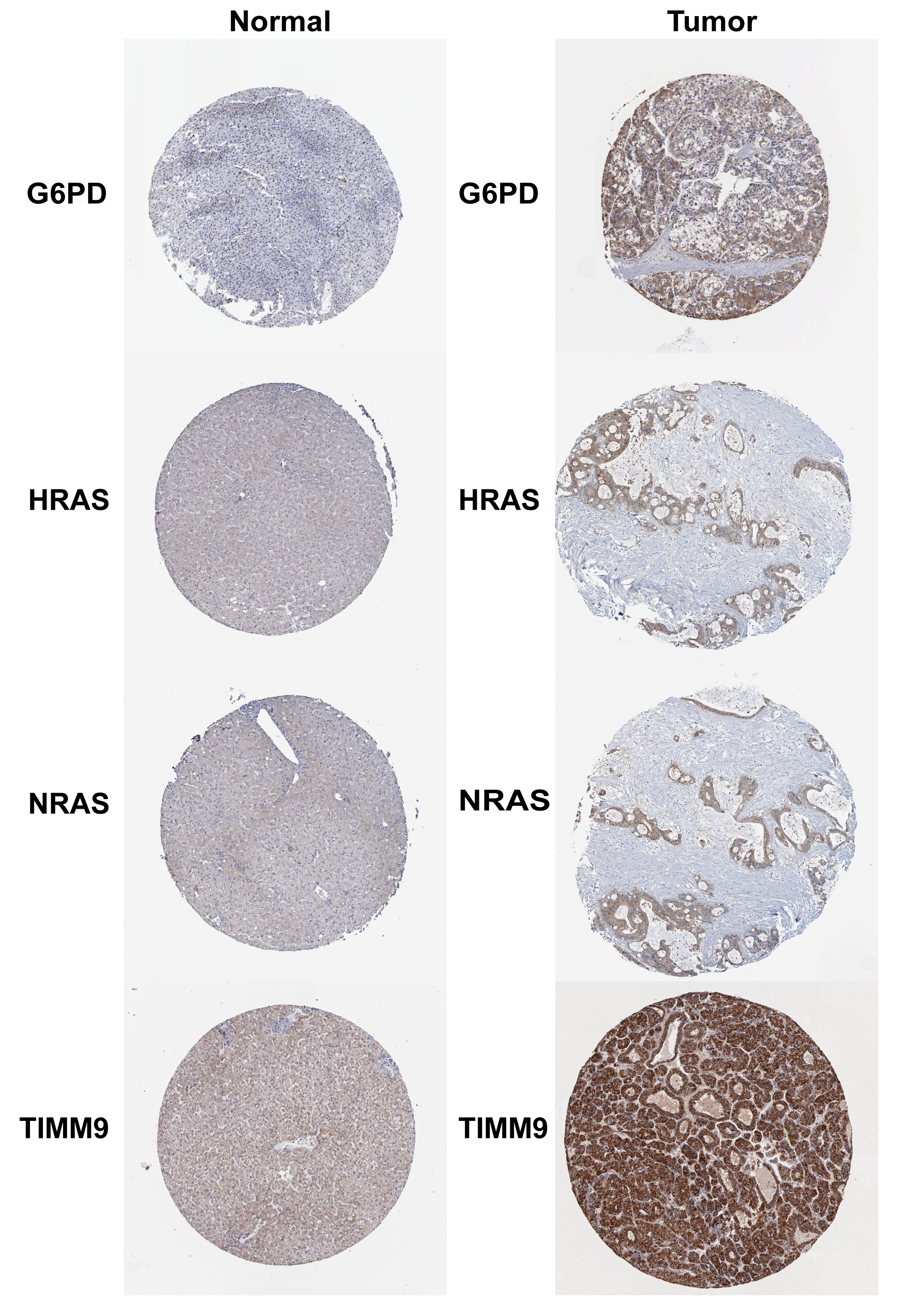

Supplement: Supplementary file 3 [file Image_3.tif]

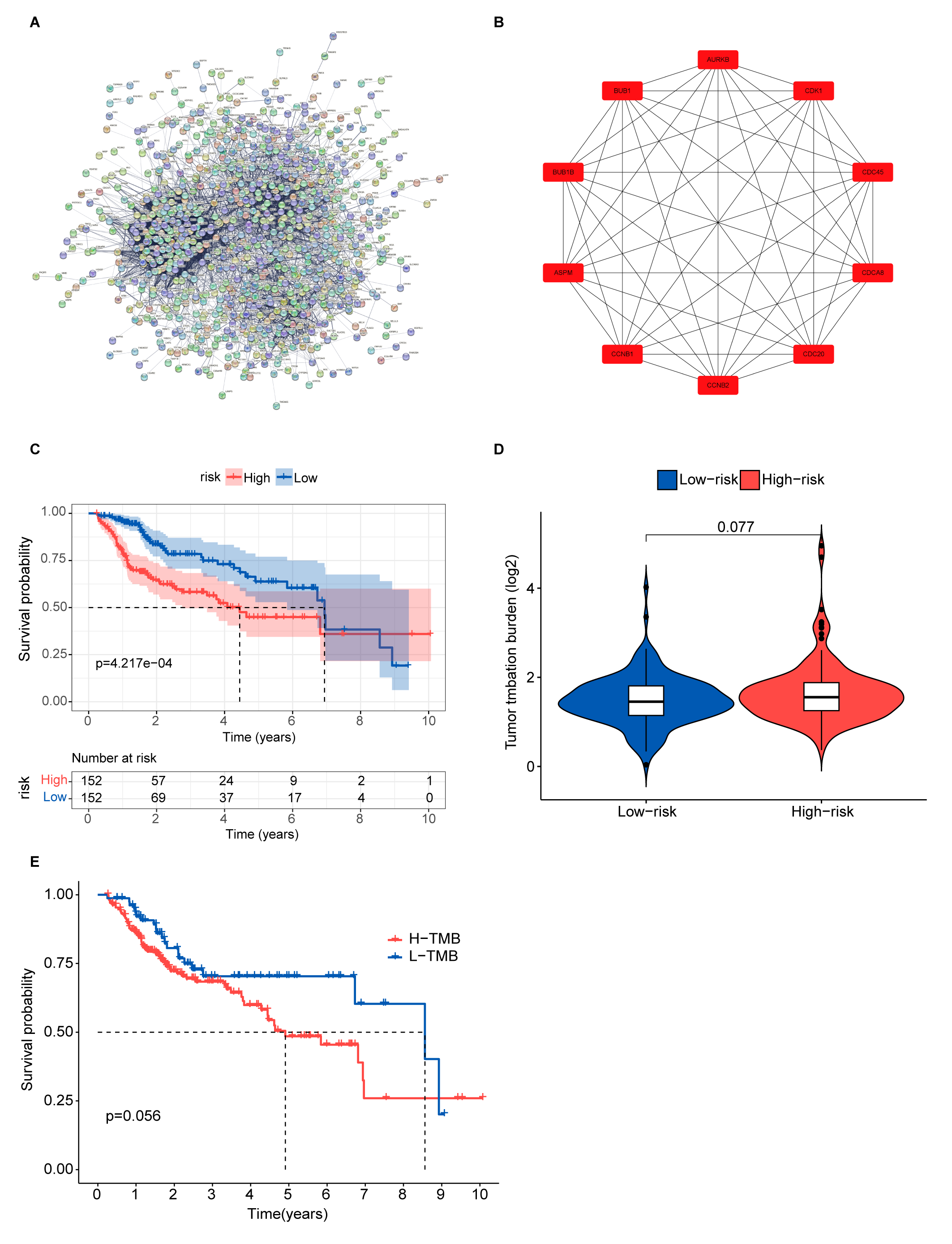

Supplement: Supplementary file 4 [file Image_4.tif]
